# Recovery of Barotrauma Injuries Resulting from Exposure to Pile Driving Sound in Two Sizes of Hybrid Striped Bass

**DOI:** 10.1371/journal.pone.0073844

**Published:** 2013-09-11

**Authors:** Brandon M. Casper, Michele B. Halvorsen, Frazer Matthews, Thomas J. Carlson, Arthur N. Popper

**Affiliations:** 1 Department of Biology, Center for Comparative and Evolutionary Biology of Hearing University of Maryland, College Park, Maryland, United States of America; 2 Pacific National Northwest Laboratory, Marine Sciences Laboratory, Sequim, Washington, United States of America; University of Windsor, Canada

## Abstract

The effects of loud sounds on fishes, such as those produced during impulsive pile driving, are an increasing concern in the management of aquatic ecosystems. However, very little is known about such effects. Accordingly, a High Intensity Controlled Impedance Fluid Filled wave Tube (HICI-FT) was used to investigate the effects of sounds produced by impulsive pile driving on two size groups of hybrid striped bass (white bass *Morone* *chrysops* x striped bass *Morone* *saxatilis* ). The larger striped bass (mean size 17.2 g) had more severe injuries, as well as more total injuries, than the smaller fish (mean size 1.3 g). However, fish in each size group recovered from most injuries within 10 days of exposure. A comparison with different species from previously published studies show that current results support the observation that fishes with physoclistous swim bladders are more susceptible to injury from impulsive pile driving than are fishes with physostomous swim bladders.

## Introduction

With the growth in development of off-shore energy sources there is increasing concern that the sounds associated with such activities have a potential negative effect on fishes [1–3]. In particular, construction of structures such as offshore wind farms, oil and gas drilling platforms, and similar activities often require impulsive pile driving which produces loud (e.g., >210 dB re 1 µPa) [3], and potentially damaging sounds. However, there are few data on the impact of impulsive sounds from pile driving and other sources (e.g., seismic air guns used in oil and gas exploration) on fishes. Moreover, most of the earlier studies were performed in the field under conditions that did not allow for controlled exposures or for quantitative analysis of their effects or where investigators could have a detailed knowledge of the received sound levels to which the fishes were exposed [1].

More recently, laboratory studies have provided new and important insight into the potential effects arising from exposure to impulsive sounds produced during pile driving on a number of species including Chinook salmon ( *Oncorhynchus* *tshawytscha* ) [4–6], lake sturgeon ( *Acipenser* *fulvescens* ), Nile tilapia ( *Oreochromis* *niloticus* ), hogchoker ( *Trinectes* *maculatus* ) [7], and the common sole ( *Solea* *soleae* ) [8]. These experiments established the first quantifiable data for evaluating sound exposure levels (SEL) that potentially result in injuries in these different species, as well as the potential for recovery from injuries in the Chinook salmon [6].

From these laboratory studies, it was also determined that the types of injuries that occur in response to pile driving exposure may differ, depending on the type of swim bladder that a fish has. Physostomous fishes (e.g. salmon, sturgeon) have a connection between the swim bladder and gut that allows the fish to gulp or expel air to change the volume of gas in the swim bladder. Being able to expel air in response to exposure to loud sounds could reduce the mass of gas and subsequent range of motion of the swim bladder, likely minimizing injuries to the swim bladder and surrounding organs.

In contrast, physoclistous fishes (e.g. tilapia) can only change the volume of gas in the swim bladder through diffusion of gasses into and out of the blood via a gas gland. This diffusion is a slow process, and based on the previously mentioned studies [4–7], fishes with physoclistous swim bladders had more severe and higher numbers of injuries compared with physostomous fishes when exposed at equal sound exposure levels. Fishes without swim bladders (i.e. hogchoker, common sole) did not show evidence of any injuries in response to pile driving exposure, at least at the SELs used in the studies [7,8].

Despite these recent studies, there still are many fundamental questions that need to be answered in order to better understand the effects of impulsive pile driving on fishes. One such question is how different sized fish of the same species respond to exposure to pile driving sounds. It has been suggested that fishes of less than 2 g are more likely to be susceptible to effects of pile driving than larger fish [1,9,10]. This hypothesis was based on studies that investigated effects of underwater explosions and suggested that smaller fish had more severe injuries than larger fish of the same species [11,12]. However, the relationship between effects of explosions vs. effects of acoustic signals is not clear, and so it is important to test this hypothesis using sound.

Another critical question is whether different species all have the potential to recover from effects of pile driving in the same way and over the same time course. Casper et al. [6] demonstrated that Chinook salmon maintained in the laboratory recover from most effects within about 10 days of exposure. However, comparable data are not available for any physoclistous species.

In order to provide data to address these questions, the present study examined effects of pile driving on hybrid striped bass (white bass *Morone* *chrysops* x striped bass *Morone* *saxatilis* ) of two different sizes; average 1.3 g and 17.2 g. This study thus tests the hypothesis that fishes less than 2 g are more susceptible to injury than larger fish when exposed to impulsive pile driving. Furthermore, the hybrid striped bass has a physoclistous swim bladder which allows for a comparison of injury and recovery responses to species with a physostomous swim bladder investigated earlier [4,5,7].

## Materials and Methods

### Ethics Statement

Experiments were conducted under supervision and approval of the Institutional Animal Care and Use Committee (IACUC) of the University of Maryland (protocol R-09-23).

### Fish Species Information

The small fish (42.3 ± 3.0 mm SL and 1.3 ± 0.3 g) were purchased in July–August, 2011 from Keo Fish Farms, Inc. (Keo, AR). Large fish (99.7 ± 15.5 mm and 17.2 ± 7.9 g) were obtained from Dr. Curry Woods (University of Maryland, MD) in December, 2010 and were originally obtained from Keo Fish Farms, Inc. (Keo, Arkansas). Later, fish of the same size as the large fish used earlier were obtained directly from Keo Fish Farms from January–October of 2011.

All fish used in the study were acclimated to the laboratory for a minimum of two weeks before being used in experiments, during which time they exhibited control of buoyancy and normal behavior. The caudal fins of the large fish were clipped for individual identification during experiments but this was not possible in the small fish due to the small sizes of their fins. Fish were maintained on a 14:10 light/dark cycle in 890 l round tanks. Animals scheduled to be used in experiments were not fed for at least three days prior to a treatment so that their digestive system would be void of food during sound exposure.

### Pile driving exposure equipment and signal presentation

**Figure 1:**
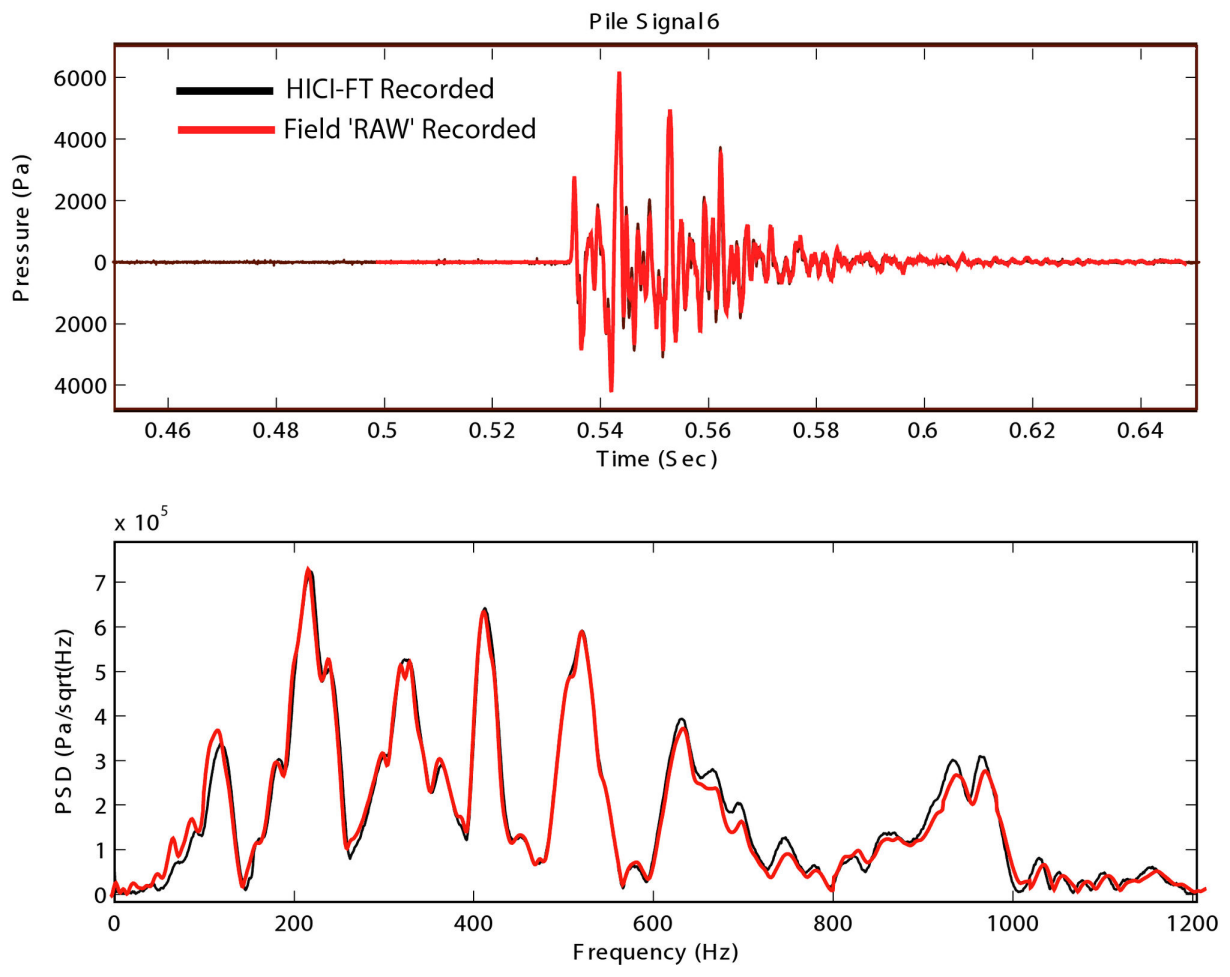
Comparison of field recording of pile driving signal with the identical signal played through the HICI-FT. The top figure shows the temporal spectrum of the signals and the bottom figure shows the frequency spectrums. PSD is power spectral density.

Pile driving exposure was conducted using the High Intensity Controlled Impedance Fluid filled wave Tube (HICI-FT; See Halvorsen et al. [4,5] for details of the equipment and its use). The HICI-FT has a water-filled cylindrical holding chamber 45 cm long and 25 cm internal diameter, with 3.81 cm-thick stainless steel walls. A rigid circular piston held by a membranous seal in the center of the steel end cap was located at either end of the tube. Each piston was connected to a moving coil shaker (Vibration Test Systems, VG-150 Vibration Generator, Model VTS 150, Aurora, OH) anchored to the end caps. The shaker motors could be driven independently in amplitude and phase to generate plane-wave pressure or velocity fields within the tube. The shakers were used to play back sounds that accurately reproduced the acoustic characteristics and sound levels of previously recorded pile driving sounds under far-field plane wave acoustic conditions (Figure 1).

Signal generation and data acquisition for the HICI-FT are described in detail in Halvorsen et al. [4,5]. The pile driving sounds used in this study were field recordings taken at 10 m from a 76.2 cm steel shell pile (outer diameter) driven using a diesel hammer at the Eagle Harbor Maintenance Facility [13]. Eight different recordings of pile driving strikes were normalized to the same SEL. Twelve repetitions of each of the eight strikes generated a file of 96 strikes that were randomized each day using MATLAB (The MathWorks, Inc., Natick, Massachusetts). That file was repeated 10 times for a 960-strike presentation. Sound presentation was controlled using LabVIEW (National Instruments Corporation, Austin, Texas). Impulsive sound levels in the tube were measured using a hydrophone mounted inside the HICI-FT (Brüel & Kjær Sound & Vibration Measurement A/S, Naerum, Denmark, Model 8103). The characteristics of the impulsive sounds were monitored to assure that the compliance of the water within the HICI-FT was not significantly altered by the number of fish placed into the device for each exposure.

The initial exposure was at a cumulative sound exposure level (SEL_cum_) of 213 dB re 1 µPa^2^·s, derived from 960 pile strikes at a single strike sound exposure level (SEL_ss_) of 183 dB re 1 µPa^2^·s (Treatment 1). SEL_ss_ (and consequently SEL_cum_) were decreased in 3 dB steps for each treatment as summarized in Table 1. From here forward, the study will refer to Treatments 1 to 5 to simplify the reference to the exposure paradigms.

**Table 1:** List of treatment metrics in terms of cumulative sound exposure level (SEL_cum_) and single strike sound exposure level (SEL_ss_).

Treatment	SEL_cum_ (dB re 1 µPa^2^·s)	SEL_ss_ (dB re 1 µPa^2^·s)	# Large HSB	# Small HSB
1	213	183	90 E/ 32 C	73 E/ 20 C
2	210	180	92 E/ 27 C	75 E/ 18 C
3	207	177	92 E/ 32 C	31 E/ 9 C
4	204	174	104 E/ 32 C	32 E/ 8 C
5	201	171	90 E/ 26 C	38 E/ 8 C

Sample sizes of hybrid striped bass (HSB) including all fish designated for recovery for treatments in which this was tested. All exposure treatments contained 960 pile strikes. E = Exposed; C = Control

### Fish exposure

The experiments used 618 (469 experimental and 149 control) large fish and 312 (249 experimental and 63 control) small fish. Control fish were subject to the identical treatment as exposed fish but without the pile driving sound.

The fresh water used to fill the HICI-FT was maintained at 18 C. Water quality was monitored throughout the duration of the study. Prior to being poured into the exposure and acclimation chambers, the water was conditioned by passing it through filtration and bioballs to maintain the gas saturation of the water at 100%. Higher gas saturations were avoided since this could result in the production of bubbles which could affect the acoustic qualities of the HICI-FT chamber as well as supersaturating the tissues of fish.

Either four large or eight small hybrid striped bass were placed into an acrylic chamber mounted around the opening of the HICI-FT exposure chamber and left to acclimate for 20 minutes. Following observations to assure the fish had maintained buoyancy control, the fish were then gently corralled into the HICI-FT chamber, the chamber closed, and the HICI‑FT rotated from the vertical to the horizontal position. Buoyancy was documented in all fishes as was done in previous studies [4–7], and it was observed that the bass always displayed neutral buoyancy. Following the completion of each treatment, fish were removed from the HICI-FT and either immediately necropsied for barotrauma assessment or returned to a recovery tank for periods of 2, 5, or 10 days before necropsy.

During the recovery periods, fish were fed on their normal schedule (three feedings per week). Following each treatment or recovery period, fish were euthanized in buffered MS-222, necropsied, and examined for external (e.g. injuries to eyes, gills, fin hematomas) and internal (swim bladder ruptures, hemorrhaging/hematomas of tissues and organs) signs of barotrauma utilizing methodology from previous studies [4–7]. Each potential injury was noted as present or not (for a detailed list of all potential barotraumas injuries see Halvorsen et al. [4]).

At all treatment levels, exposure and necropsy evaluation was conducted at day 0 for both size classes. Recovery from injuries was evaluated at days 2, 5, and 10 for all of the large fish while only treatments 1 and 2 were evaluated for the small fish due to an insufficient number of animals.

### Evaluation of barotrauma injuries

Evaluation of the severity of injuries in response to the pile driving stimuli was accomplished following the Response Weighted Index (RWI) equation used in previous studies [4,5,7]:

RWI=∑(Injury∗Weight) (1)

In this equation, injuries were assigned to weighted trauma categories based on the physiological impact of the fish: *Mortal*, *Moderate*, or *Mild*. The *Mortal* trauma category, weighted 5, included injuries that were severe enough to lead to death (in this study these injuries were ruptured swim bladder and renal hemorrhaging). The *Moderate* trauma category, weighted 3, included injuries likely to have an adverse impact on fish health but might not lead directly to mortality (in this study these injuries were swim bladder herniations, damage to the gall bladder, intestinal hemorrhaging, and all hematomas). Finally, *Mild* trauma category, weighted 1, referred to injuries of minimal to no physiological cost to fish (there were no examples of this injury observed in this study but examples would be fin hematomas). Weighting allowed complex and variable data to be reduced to a single value for each fish. The RWI value is the sum of the presence of each injury multiplied by the trauma weight assigned to each injury type.

In fish designated for recovery periods, injury analysis was accomplished using the injury evaluation index established by Casper et al. [6]. This analysis was used because some injuries (i.e. many of the hematomas) appeared at higher frequencies during the later post-exposure days. Furthermore, the probability of detection of injuries was inconsistent with the probability of the occurrence of these injuries. The injury index was calculated by determining the ratio of frequency of occurrence of injuries for each individual injury observed in the sample of fish at each time point (i.e. number of times an injury appears divided by total number of fishes in a treatment that could potentially incur the injury) and multiplied by 100, and then summed over all injury types observed in the sample.

Injury Index=∑(Injury Occurrence Ratio∗100) (2)

### Statistical Analysis

A 2-way ANOVA (SigmaPlot 11, SYSTAT Software, Inc.) was used to evaluate the two dependent variables, RWI values and number of injuries observed between fish sizes and treatment levels at day 0 post exposure. A 3-way ANOVA was used to evaluate number of injuries as related to fish size, treatment, and days post exposure recovery period. The 2-way ANOVA test was also used to compare the RWI and number of injuries at each treatment level between the large hybrid striped bass with results collected previously from other fish species (Halvorsen et al. 2011, 2012a,b). A post hoc Tukey test was used to evaluate any differences within and between sizes and species in terms of both RWI values (for analyses at day 0 post exposure) as well as number of injuries observed. An α of 0.05 was used for all statistical tests. All statistical information is presented in Tables 2-6.

**Table 2:** 2 Way ANOVA analysis of RWI values for interaction of the different sizes of hybrid striped bass (HSB) and treatment levels at day 0 analysis.

**RWI**	**DF**	**SS**	**MS**	**F**	**P**
Fish Size	1	6839.039	6839.039	660.517	<0.001
Treatment	4	19205.124	4801.281	463.710	<0.001
Fish Size x Treatment	4	10498.281	2624.570	253.482	<0.001
Residual	315	3261.531	10.354		
Total	324	39025.025	120.448		

See File S1 for post-hoc analysis.

**Table 3:** 2 Way ANOVA analysis of number of injuries observed for interaction of the different sizes of hybrid striped bass (HSB) and treatment levels at day 0 analysis.

**# of Injuries Observed**	**DF**	**SS**	**MS**	**F**	**P**
Fish Size	1	13.917	13.917	24.392	<0.001
Treatment	4	276.341	69.085	121.085	<0.001
Fish Size x Treatment	4	17.250	4.313	7.559	<0.001
Residual	315	179.724	0.571		
Total	324	486.012	1.500		

See File S1 for post-hoc analysis.

**Table 4:** 3-way ANOVA analyses of number of injuries observed for interactions between sizes of hybrid striped bass (HSB), each day post exposure, and treatments.

**# of Injuries Observed**	**DF**	**SS**	**MS**	**F**	**P**
Fish Size	1	13.917	13.917	24.392	<0.001
Treatment	4	276.341	69.085	121.085	<0.001
Days Post Exposure	3	54.809	18.270	38.083	<0.001
Fish Size x Treatment	4	17.250	4.313	7.559	<0.001
Fish Size x Days Post Exposure	3	33.329	11.110	23.158	<0.001
Treatment x Days Post Exposure	12	64.376	5.365	11.183	<0.001
Fish Size x Treatment x Days Post Exposure	3	4.767	1.589	3.312	.020
Residual	315	179.724	0.571		
Total	324	486.012	1.500		

Comparison between fish sizes was only conducted for treatments 1 and 2.See File S1 for post-hoc analysis.

**Table 5:** 2-way ANOVA analysis of RWI values for interactions between the large hybrid striped bass and previously collected data of different species [4,5,7] and treatment levels.

**RWI**	**DF**	**SS**	**MS**	**F**	**P**
Species	3	12946.210	4315.403	498.696	<0.001
Treatment	3	21873.760	7291.253	842.592	<0.001
Species x Treatment	9	11335.663	1259.518	145.552	<0.001
Residual	476	4119.002	8.653		
Total	491	49394.429	100.600		

See File S1 for post-hoc analysis.

**Table 6:** 2-way ANOVA analysis of number of injuries for interactions between the large hybrid striped bass and previously collected data of different species [4,5,7] and treatment levels.

**# of Injuries Observed**	**DF**	**SS**	**MS**	**F**	**P**
Species	3	64.558	21.519	33.551	<0.001
Treatment	3	371.691	123.897	193.168	<0.001
Species x Treatment	9	32.706	3.634	5.666	<0.001
Residual	476	305.305	0.641		
Total	491	770.797	1.570		

See File S1 for post-hoc analysis.

## Results

All fish survived exposure to pile driving sounds in the HICI-FT and no animals died until being euthanized for necropsy. Fish were observed several times each day over the course of the recovery and there were no apparent differences in feeding, swimming, buoyancy control, or other behaviors between experimental and control animals.

**Figure 2:**
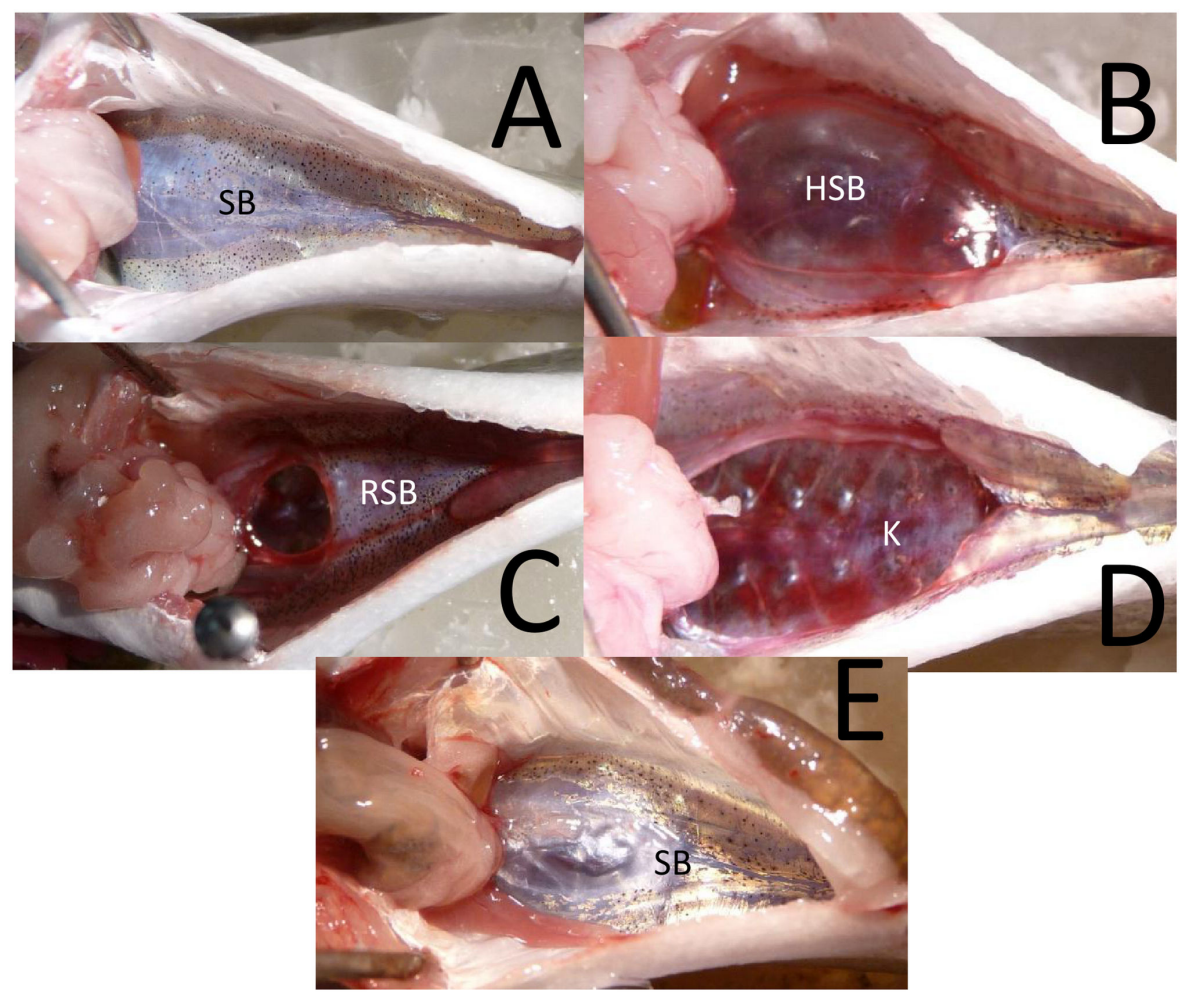
Examples of injuries in the larger hybrid striped bass. **A**. Control fish showing a healthy swim bladder (SB). **B**. Fish with herniated swim bladder (HSB). **C**. Fish with ruptured swim bladder (RSB). **D**. Fish with kidney hemorrhaging (K). E. Fish with fully healed swim bladder as evidenced by the white scar tissue on the swim bladder (SB). All photos are the ventral view of the fish with anterior to the left.

**Figure 3:**
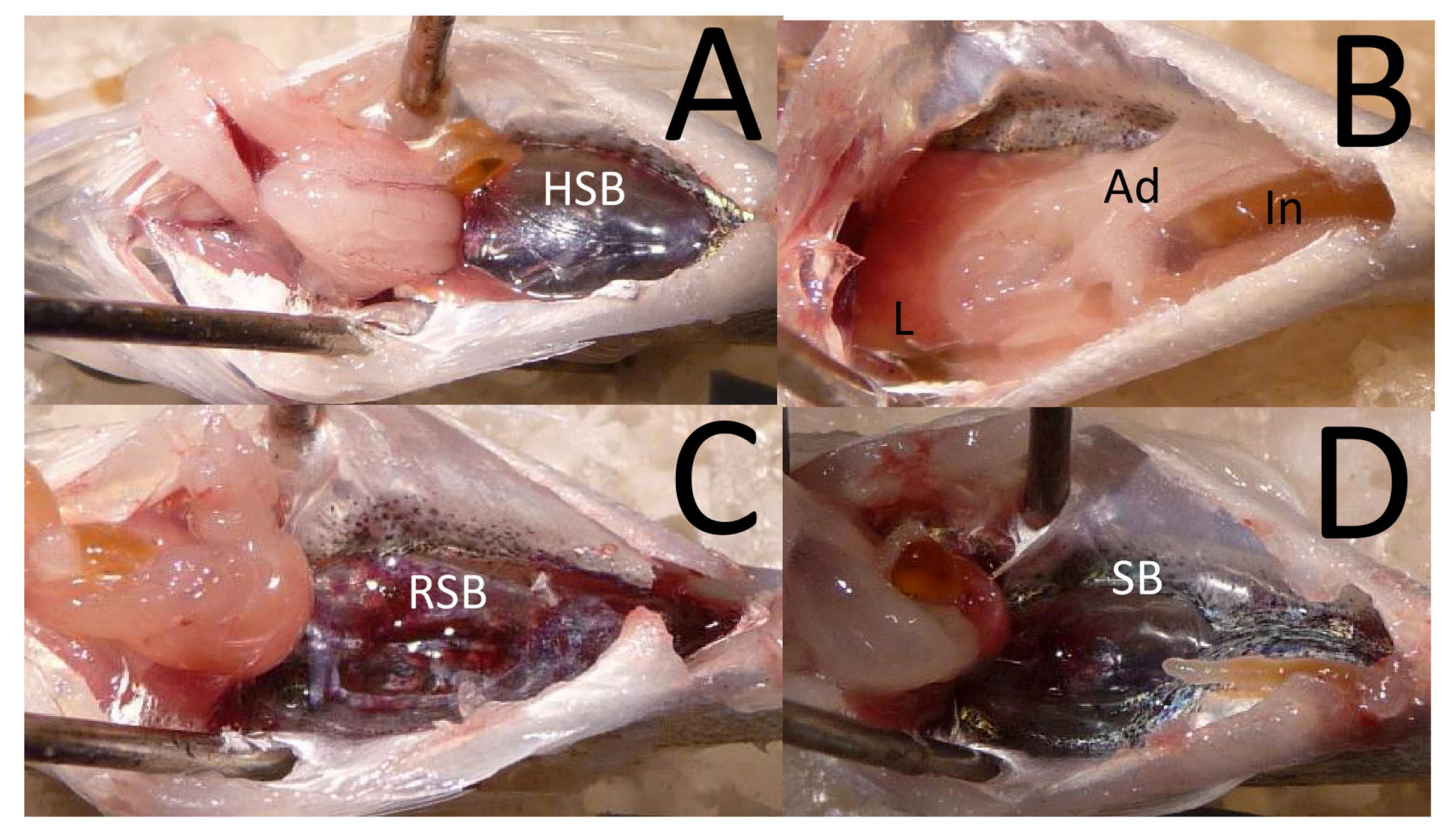
Examples of injuries in the smaller hybrid striped bass. **A**. Fish with a herniated swim bladder. **B**. Fish with a liver (L) hematoma. **C**. Fish with a ruptured swim bladder (RSB). **D**. Fish with fully healed swim bladder as evidenced by the white scar tissue on the swim bladder (SB). Organs visible within the pictures include adipose tissue (Ad) and intestine (In). All photos are the ventral view of the fish with anterior to the left.

**Figure 4:**
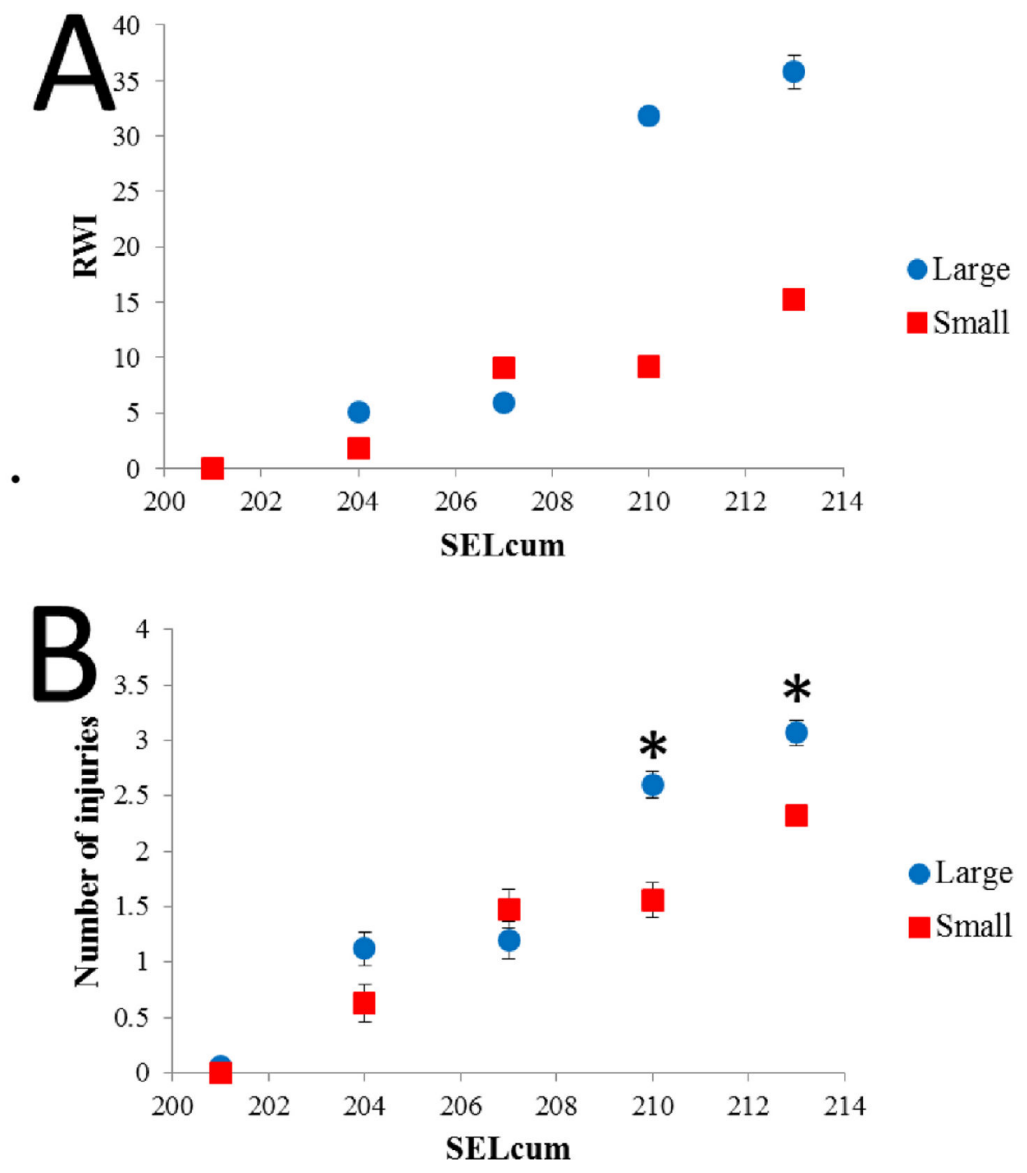
Analysis of injuries for fish sample at day 0 post exposure. **A**. RWI values at day 0 post exposure of each treatment in terms of SEL_cum_ for the two different sizes of hybrid striped bass. **B**. Number of injuries observed at day 0 post exposure of each treatment in terms of SEL_cum_ for the two different sizes of hybrid striped bass. Error bars signify standard deviation values.

Although the same nine types of injuries were observed in large and small fish (Figures 2, 3), the large fish had significantly higher RWI values (Table 2) and number of observed injuries (Table 3) then the small fish (Figure 4) and there was a significant interaction between fish size and treatment. There were no injuries in the control fish (Figure 2).

The injuries in sound-exposed animals included ruptured swim bladder and renal hemorrhage which are both classified as *Mortal* injuries. There were also swim bladder herniation, swim bladder hematomas, gall bladder hemorrhage, intestinal hemorrhage, hepatic hematomas, gonadal hematomas, and adipose hematomas, all of which are classified as *Moderate* injuries (Figures 2, 3). There were no external injuries observed in any fish.

### Effects immediate post exposure

There were significant differences correlated with treatment level in the large fish for both the calculated RWI values (Table 2) (Figure 4) and actual number of injuries at day 0 (Table 3) (Figure 4). Both calculated RWI and actual number of injuries decreased from Treatment group 1 to 5.

**Figure 5:**
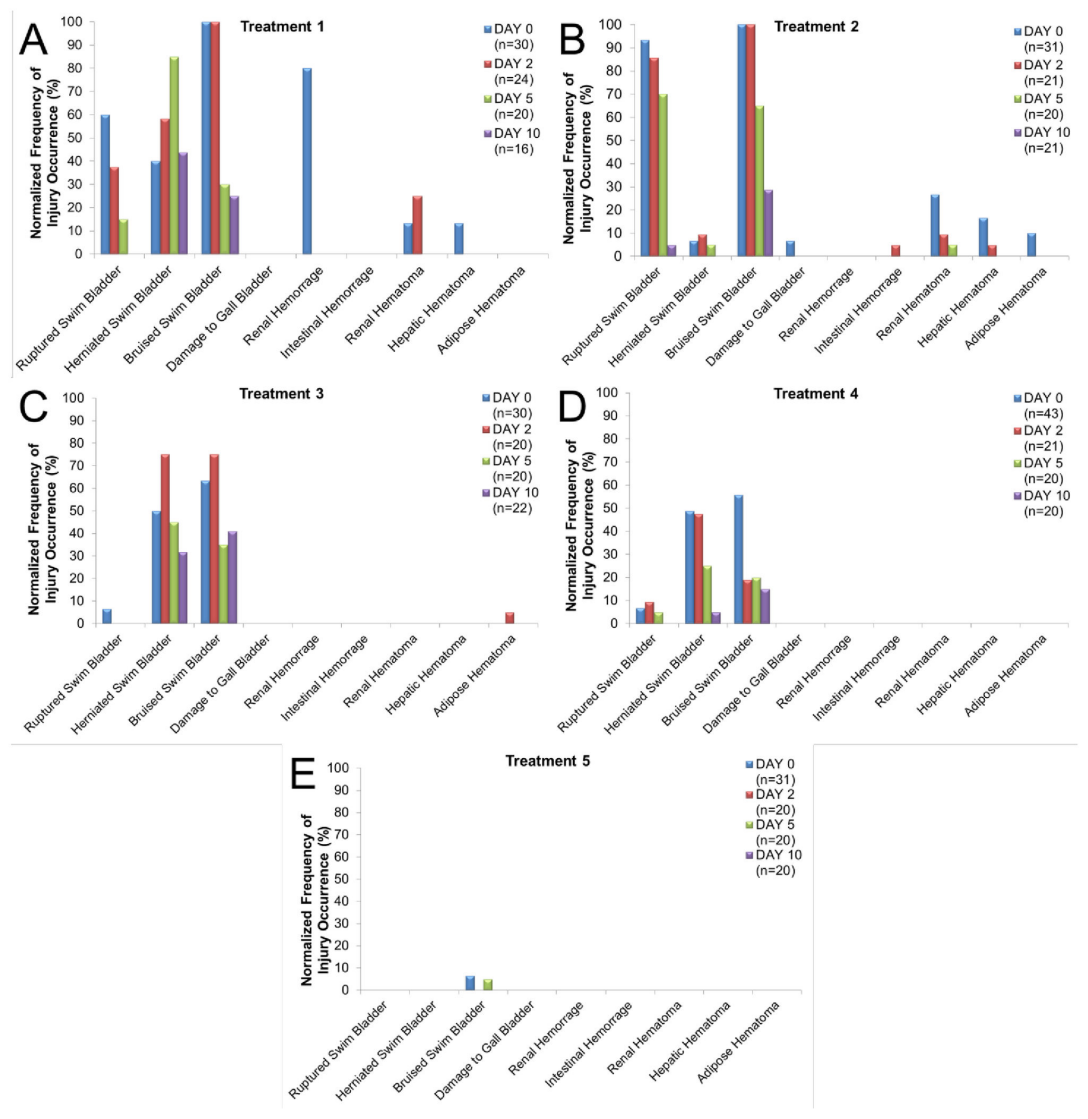
Normalized frequency of occurrence of each injury in large hybrid striped bass for each day post exposure within each Treatment.

In terms of RWI values, all treatments were significantly different from one another except for treatments 3 and 4 (Table 2). In contrast, there was no significant difference in number of injuries observed between treatments 1 and 2 and treatments 3 and 4 (Table 3). The highest rates of the *Mortal* injuries were found in treatments 1-2 for ruptured swim bladders and treatment 1 for kidney hemorrhaging (Figure 5). *Moderate* injuries of the swim bladder were fairly common through treatments 1-4 and for other anatomical structures through treatments 1-2, though by treatment 5 there were almost no injuries of any kind (Figure 5).

**Figure 6:**
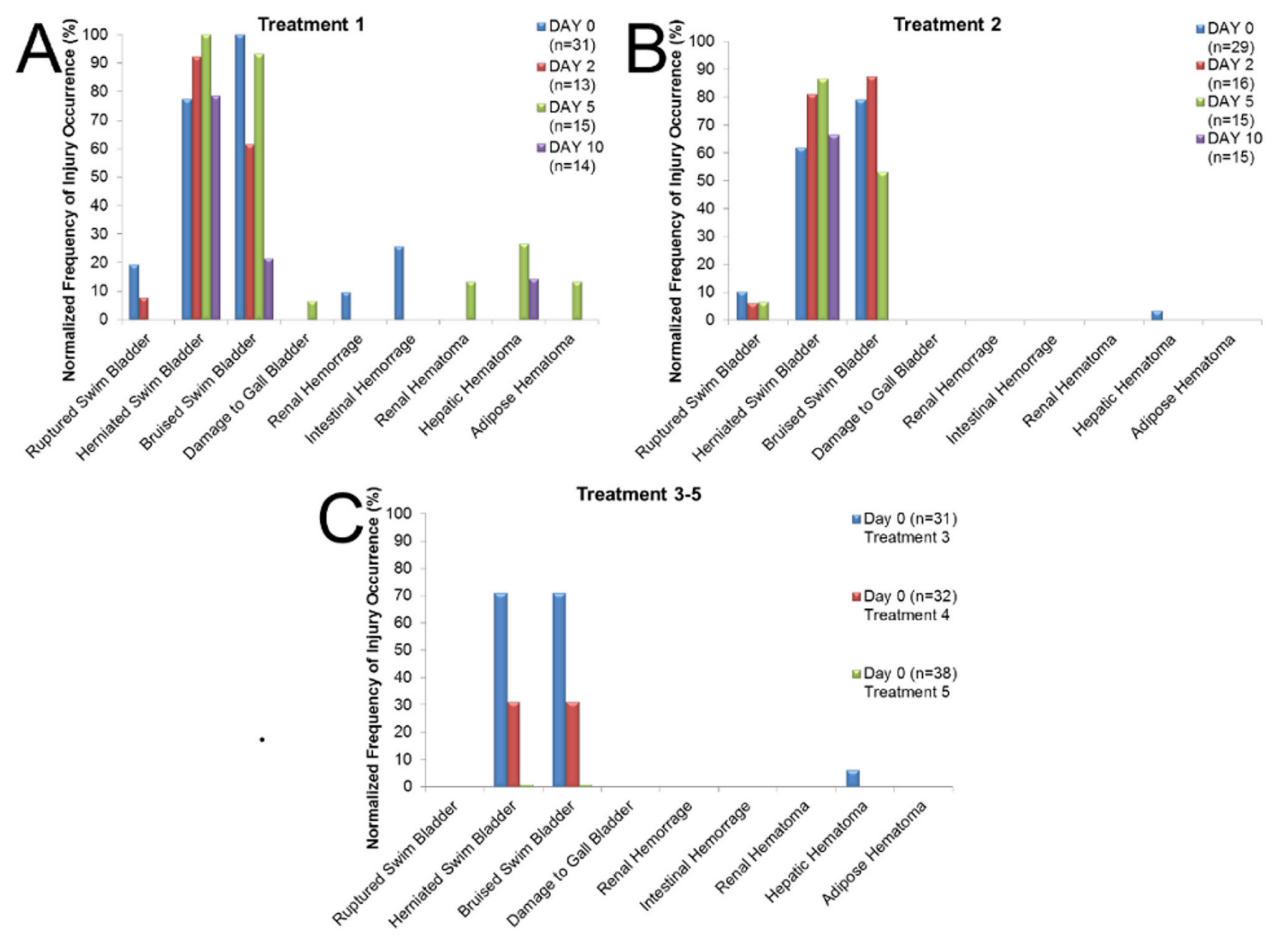
Normalized frequency of occurrence of each injury for small hybrid striped bass for each day post exposure within each Treatment.

There were significant differences for small fish among treatment levels for both RWI values (Table 2) and number of injuries at day 0 (Table 3) (Figure 4). There was a significant decrease in RWI values between treatments 1 and 2 as well as treatments 3 and 4 (Table 2). Furthermore, there was a significant difference between all treatments except for treatments 2 and 3 for number of injuries observed (Table 3). Herniated and bruised swim bladders were the most common injuries in treatments 1-4 with less than 30% being ruptured swim bladders, kidney and intestinal hemorrhages, and liver hematomas (Figure 6). There were no injuries observed at day 0 during treatment 5 (Figure 6).

### Recovery from injuries

All interaction terms in the ANOVA model had significant effects and there were significant main effects for fish size, treatment and days post exposure (Table 4). There was a significant difference in recovery for large fish in the number of injuries between treatment levels over post exposure sample times (Table 4). This included a significant decrease in the number of injuries observed between post exposure days 0, 2, and 5 in treatment 1, between days 2 and 10 in treatment 2, and between days 2 and 5 in treatment 3 (Table 4) (Figure 5). All other days and treatments did not significantly differ from one another, although treatments 1-4 showed a general decrease in number of injuries from days 0 through 10. Injury index values also showed a decreasing trend throughout most of the treatments, with no trend at treatment 5 where there were almost no injuries observed at any of the days post exposure.

Damage to the swim bladder in the large fish was the most commonly observed injury in treatments 1-4 during the recovery analysis (Figure 5). In most cases, the highest frequency of injuries occurred at days 0 and 2. However, in treatment 1, herniated swim bladders peaked at day 5. By day 10 post exposure, the frequency of occurrence of all injuries were less than 50%, and most had 0% occurrence. Many of the fish showed scarring of the swim bladder tissue indicating evidence of healing of swim bladder damage (Figure 2).

Small fish showed a significant difference in the number of injuries observed between treatments and days post exposure (Table 4). By treatment, there was a significant difference in number of injuries observed between all days for treatment 1 and between days 5 and 10 for treatment 2 (Table 4) (Figure 6). The injury index values at treatment 1 showed a decrease from day 0 to 2, followed by an increase at day 5 and then a decrease at day 10. Within treatment 2 there was little difference in injury index value between days 0-5, with a large decrease at day 10.

Injuries to the swim bladder were most commonly observed in days 2, 5, and 10 post exposure, with ruptured swim bladders in less than 10% of the treatments (Figure 6), a finding similar to that in the large fish. A similar increase was observed in the frequency of herniated swim bladders from days 0-5, but this then decreased at day 10 in treatments 1 and 2 (Figure 6).

Non-swim bladder injuries were only observed in treatment 1 and in less than 30% of the small fish. With the exception of swim bladder and liver hematomas in treatment 1 and herniated swim bladders in treatments 1 and 2, all other injuries appeared to be healed by day 10. Many small fish also showed evidence of scar tissue on their swim bladders (Figure 3).

When comparing sizes, the larger fish had significantly higher RWI values (Table 2) and number of injuries observed for day 0 (Table 3) within treatments 1, 2 and in number of injuries observed in treatment 4 (Figure 4). All other treatments did not differ significantly between fish size.

Post exposure recovery analysis was conducted for the two sizes of fish for treatments 1 and 2. A significant difference was found in the number of injuries at both treatment levels (Table 4). Within treatment 1, there was a significant difference at days 0, 2, and 5 between large and small fish and an increase in the mean numbers of injuries in the large fish at days 0 and 2, while the small fish had more injuries at days 5 and 10 (Figures 5, 6). Treatment 2 followed the same pattern, with the large fish having more injuries at days 0 and 2 and the small fish having more injuries at days 5 and 10, though only day 0 was statistically different (Figures 5, 6).

## Discussion

### Size analyses

While most fisheries biologists would not consider the larger striped bass used here to be representative of even medium sized animals in hatcheries or in the wild, the size of fish that could be tested was limited by the size of the HICI-FT chamber. At the same time, since the goal of this portion of the study was to test the hypothesis that fish less than 2 g are more susceptible to injury than fish greater than 2 g, the absolute size of the animals was less important than relative size.

The origin of the hypothesis [9] that fish smaller than 2 g are more susceptible to injury than fish greater than 2 g stems from the response of different size fishes to rapid decompression caused by impulsive sound generated by in-water explosions [11]. Yelverton et al. [11] found a correlation with body size and received blasting sound impulse level, with large fishes experiencing lower mortality than small fishes. While the characteristics of the impulsive sound generated by underwater explosions are not the same as those generated by pile driving, the two sources do share some temporal and spectral characteristics including rapid onset of the sounds [1,14]. In contrast to the explosive data [11] and the hypothesis proposed by Carlson et al. [9], results from the current study on impulsive pile driving show less damage in small hybrid striped bass with average weights of 1.8 g than in larger fish of 17.1 g. The large fish had higher RWI values (Figure 4) and number of injuries at the loudest treatments levels (1 and 2) at day 0 compared to the small fish (Figure 5). This suggests that the large hybrid striped bass sustained more severe injuries based on the RWI values as well as sustaining a greater total numbers of injuries.

While it is hard to know the cause of the increased injury in larger fish versus small fish, one possible explanation could be the difference in swim bladder resonance. There is evidence that exposing mice to sound energy focused at the lung resonance can cause injury to the lungs and surrounding tissues [15]. If the spectral energy levels of the pile driving signals peaked at frequencies centered around the large hybrid striped bass swim bladder resonance, then this would likely result in more swim bladder motion and increased numbers and severity of injuries. However, without knowing the swim bladder resonance of either of the size groups of hybrid striped bass, this is purely conjecture. Alternatively, there may be differences in the degree of contact between the swim bladder and the surrounding tissues, resulting in the degree that the swim bladder strikes other tissues. Other alternative hypotheses might be the thickness of the swim bladder walls in different sized fishes and/or the resilience of older tissues in larger fish to damage. Clearly, more extensive testing over a larger range of fish sizes and species is needed to fully understand this issue since it has relevance in the development of guidelines to protect fish from harm by impulsive sounds.

### Recovery Analysis

In large striped bass the basic trend was a decrease in number of injuries at each post exposure time point for treatments 1 and 2, with an average of less than one injury for each fish by day 10 (Figure 5). Trends for the small fish in terms of recovery were not as clear. Over the post exposure days in treatment 1 the numbers of injuries decreased, increased, and then decreased again (Figure 6). This increase occurred from day 2-5 and appeared to be caused by hematomas on the liver, kidney, and adipose tissue as well as by an increased occurrence of swim bladder hematomas appearing sometime after day 2 (Figure 6). These injury occurrence patterns suggest delayed injury appearance in small fish, and that, as a consequence, small fish need more time to recover from injuries sustained by the impulsive pile driving. As in the case of investigation of susceptibility of larger versus smaller fish to injury from impulsive sound, investigation of injury recovery would also benefit from studies across a greater size range of fish and species. Also important will be experimental designs that clarify the indicated delay in development of some types of injuries, such as herniated swim bladders, post exposure.

### Comparison of species

The effects and recovery of the large hybrid striped bass were compared with three previously tested species exposed to impulsive sound using the HICI-FT. For this comparison, it was possible to use necropsy data at day 0 from treatments 1-4 of this study and compare to results from identical treatment exposures of two physostomous species, the lake sturgeon and Chinook salmon, and one physoclistous species, the Nile tilapia [4,7].

**Figure 7:**
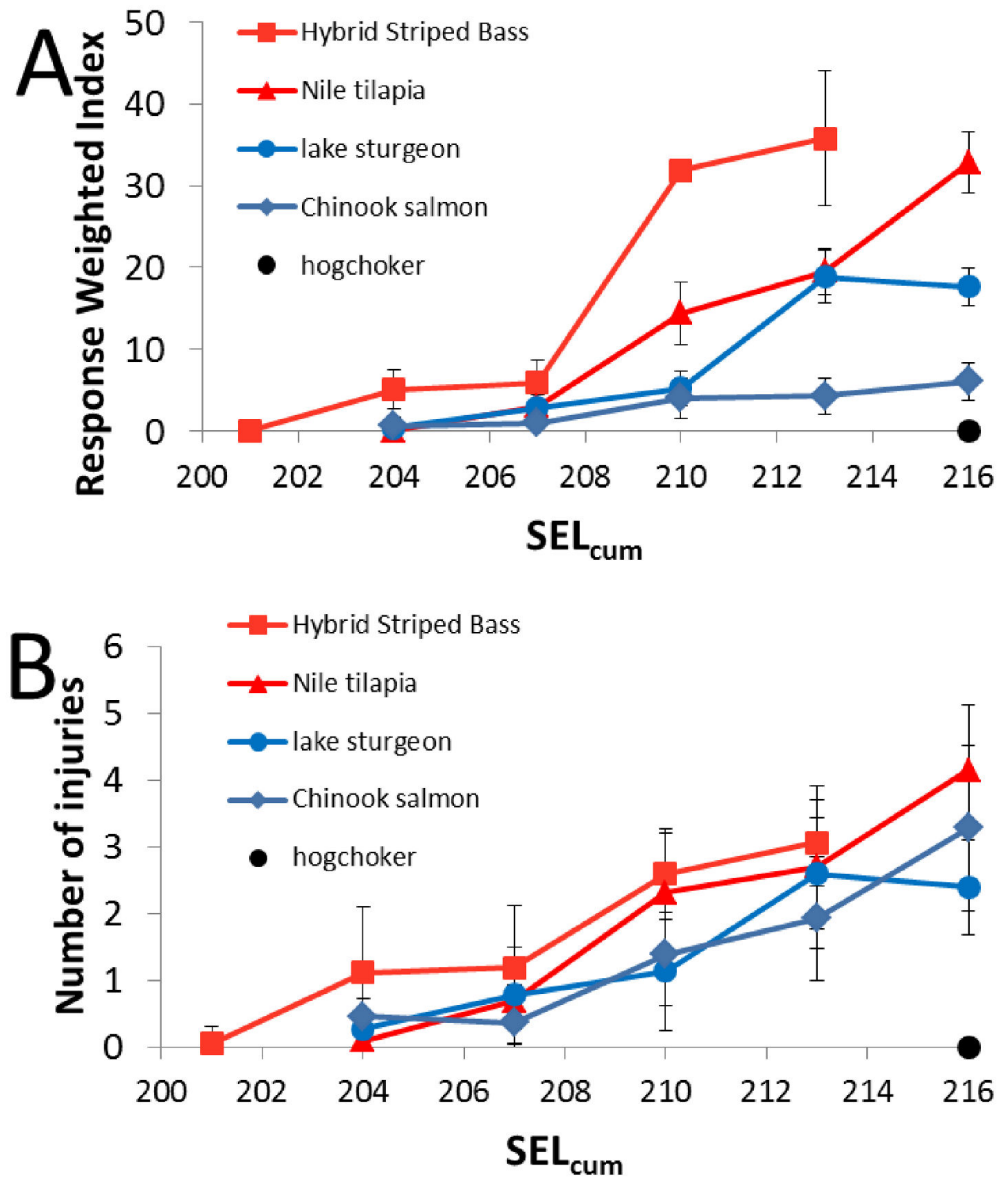
Comparison of pile driving exposure between species. **A**. RWI values at day 0 post exposure of each treatment in terms of SEL_cum_ for five species tested using the HICI-FT. **B**. Number of injuries observed at day 0 post exposure of each treatment in terms of SEL_cum_ for five species tested using the HICI-FT. The hybrid striped bass and Nile tilapia (Halvorsen et al. 2012b) are in red signifying fishes with physoclistous swim bladders. The Chinook salmon (Halvorsen et al. 2011, 2012a) and the lake sturgeon (Halvorsen et al. 2012 b) are in blue signifying fishes with physostomous swim bladders. The hogchoker (Halvorsen et al. 2012b) in black signifies a fish with no swim bladder. Error bars signify standard deviation values.

When comparing injuries, there was a large difference in responses between the hybrid striped bass and physostomous species. The hybrid striped bass had significantly higher RWI values (Table 5) and number of injuries observed (Table 6) within all treatment levels than the Chinook salmon (Figure 7). When comparing the hybrid striped bass with the lake sturgeon, the hybrid striped bass had significantly higher RWI values at all treatment levels (Table 5) as well as significantly more injuries for treatments 2 and 4 (Table 6) (Figure 7).

These results are not surprising when the swim bladder physiology is considered. Physostomous species can expel gas from their swim bladders via the pneumatic duct connection with their gut, thereby potentially rapid reduction in swim bladder pressure at exposure to impulsive sound, thereby decreasing the extent of movement of the swim bladder walls against the surrounding tissues, resulting in less hematoma. In contrast, fishes with physoclistous swim bladders, including the hybrid striped bass and Nile tilapia, can only remove gas from the swim bladder via diffusion through a gas gland, a slow process that would not allow pressure reduction during impulsive pile driving.

It is also possible that other physiological factors not related to the fullness of the swim bladder at exposure, such as biomechanical properties of tissues, contribute to the apparent higher susceptibility to more severe and/or greater numbers of barotrauma injuries for larger fish and fish of different species. For example, the striped bass also showed a higher sensitivity to pile driving when compared to the Nile tilapia with significantly higher RWI values (Table 5), though number of injuries observed was not different for treatments 1-3 (Table 6) (Figure 7). While it might be expected that results would be more similar since both species are physoclistous, the swim bladder wall appeared thicker in the Nile tilapia than in hybrid striped bass (Casper, personal observation), possibly resulting in less damage to the chamber walls and less movement due to increased biomechanical stiffness. However, swim bladder wall thickness and other tissue properties were not measured in these studies, but it was more difficult to cut through the Nile tilapia swim bladder. A future study could include swim bladder tissue property variables among different physoclistous species to bring biomechanical considerations into assessment of the response of fishes to impulsive sound.

### Implications for fishes exposed to impulsive pile driving

The results of this study further support the idea that fishes with a physoclistous swim bladder are more susceptible to impulsive sound injuries at equivalent SEL than their physostomous counterparts while not explaining the mechanisms for the differences [7]. At the same time, the results also demonstrate that the levels of sounds which start to elicit physiological injury are approximately similar in all species studied to date (Figure 7), and well above the levels of 187 dB re 1 µPa^2^·s SEL_cum_ for fishes above 2 g and 183 dB re 1 µPa^2^·s SEL_cum_ for fishes below 2 g currently being used as interim criteria [10,16]. In fact, the HICI-FT creates a worst-case scenario since the fish cannot avoid exposure to a high number of impulsive sounds by moving away. If fishes in the wild respond to loud sounds by moving away, their likelihood of being exposed to high cumulative sound levels is low. Additional experimentation are needed to determine the minimum number of impulsive sound exposures (i.e. pile strikes) in terms of the single and cumulate energy in impulsive sounds that result in the onset of injury

To date, two species (Chinook salmon and hybrid striped bass) have been evaluated for recovery from injuries incurred by exposure from impulsive sound. Casper et al [6] showed that by day 10 most Chinook salmon had either completely healed (quieter treatment) or had an average of less than 2 injuries which were mostly hematomas (louder treatment). The trend of healing was even more apparent in the hybrid striped bass, such that by day 10 in both size groups the average number of injuries was about one for all treatments. Furthermore, in many cases, both size groups of hybrid striped bass showed evidence of healing as judged by scarring on the surface of the swim bladder where a herniation or rupture likely had occurred. This finding of healing in two species with very different types of swim bladder physiology leads to the suggestion that all fishes are capable of healing within a reasonable length of time following exposure to impulsive sounds. However, as noted earlier [6], recovery was within a laboratory setting and did not involve stressors such as predators or having to search for food. Therefore, while the demonstration of the capability of recovery is very important, the results may not be indicative of what happens to wild fishes.

## Supporting Information

File S1 **Contains Tables S1-S5.**
(DOCX)
