# Supplementary material for: Recovery of Barotrauma Injuries Resulting from Exposure to Pile Driving Sound in Two Sizes of Hybrid Striped Bass
Source: PLoS One. 2013 Sep 11;8(9):e73844. doi: 10.1371/journal.pone.0073844 (PMC3770664; doi:10.1371/journal.pone.0073844)
Supplement: File S1 — Contains Tables S1-S5. (DOCX) [file pone.0073844.s001.docx]

**Supplementary Material**

**Table S1** Post-hoc Tukey test comparing individual treatments among **A)** large fish, **B)** small fish, and **C)** between sizes, from the 2 Way ANOVA analysis of RWI values for interaction of the different sizes of hybrid striped bass (HSB) and treatment levels at day 0 analysis.

**A.**

| **Large Fish x Treatment** | **Diff of Means** | **P Values** |
| --- | --- | --- |
| 1 vs. 2 | 3.489 | <0.001 |
| 2 vs. 3 | 27.865 | <.001 |
| 3 vs. 4 | 0.602 | 0.935 |
| 4 vs. 5 | 2.619 | 0.005 |

**B.**

| **Small Fish x Treatment** | **Diff of Means** | **P Values** |
| --- | --- | --- |
| 1 vs. 2 | 2.594 | 0.016 |
| 2 vs. 3 | 0.438 | 0.985 |
| 3 vs. 4 | 2.537 | 0.014 |
| 4 vs. 5 | 1.818 | 0.126 |

**C.**

| **Both Sizes x Treatment** | **Diff of Means** | **P Values** |
| --- | --- | --- |
| 1 | 20.212 | <0.001 |
| 2 | 26.295 | <0.001 |
| 3 | 1.131 | 0.170 |
| 4 | 0.803 | 0.281 |
| 5 | 0.002 | 0.998 |

**Table S2** Post-hoc Tukey test comparing individual treatments among **A)** large fish, **B)** small fish, and **C)** between sizes, from the 2 Way ANOVA analysis of number of injuries observed for interaction of the different sizes of hybrid striped bass (HSB) and treatment levels at day 0 analysis.

**A.**

| **Large Fish x Treatment** | **Diff of Means** | **P Values** |
| --- | --- | --- |
| 1 vs. 2 | 0.467 | 0.117 |
| 2 vs. 3 | 1.400 | <0.001 |
| 3 vs. 4 | 0.084 | 0.990 |
| 4 vs. 5 | 1.052 | <0.001 |

**B.**

| **Small Fish x Treatment** | **Diff of Means** | **P Values** |
| --- | --- | --- |
| 1 vs. 2 | 0.771 | <0.001 |
| 2 vs. 3 | 0.068 | 0.997 |
| 3 vs. 4 | 0.878 | <0.001 |
| 4 vs. 5 | 0.606 | 0.007 |

**C.**

| **Both Sizes x Treatment** | **Diff of Means** | **P Values** |
| --- | --- | --- |
| 1 | 0.744 | <0.001 |
| 2 | 1.048 | <0.001 |
| 3 | 0.284 | 1.421 |
| 4 | 0.510 | 0.004 |
| 5 | 0.065 | 0.726 |

**Table S3** Post-hoc Tukey test comparing individual treatments among **A)** large fish, **B)** small fish, and **C)** between sizes, from the 3 Way ANOVA analysis of number of injuries observed for interaction of the different sizes of hybrid striped bass (HSB), each day post exposure, and treatment levels.

**A.**

| **Fish Size** | **Number of Injuries** | **Day Post Exposure** | **Diff of Means** | **P Values** |
| --- | --- | --- | --- | --- |
| Large HSB | Treatment 1 | Day 0-2 | 0.858 | <0.001 |
|  |  | Day 2-5 | 0.908 | <0.001 |
|  |  | Day 5-10 | 0.612 | 0.035 |
|  | Treatment 2 | Day 0-2 | 0.457 | 0.082 |
|  |  | Day 2-5 | 0.693 | 0.006 |
|  |  | Day 5-10 | 1.117 | <0.001 |
|  | Treatment 3 | Day 0-2 | 0.350 | 0.277 |
|  |  | Day 2-5 | 0.750 | 0.003 |
|  |  | Day 5-10 | 0.073 | 0.986 |
|  | Treatment 4 | Day 0-2 | 0.354 | 0.200 |
|  |  | Day 2-5 | 0.262 | 0.602 |
|  |  | Day 5-10 | 0.300 | 0.498 |
|  | Treatment 5 | Day 0-2 | 0.065 | 0.987 |
|  |  | Day 2-5 | 0.050 | 0.996 |
|  |  | Day 5-10 | 0.050 | 0.996 |

**B.**

| **Fish Size** | **Number of Injuries** | **Day Post Exposure** | **Diff of Means** | **P Values** |
| --- | --- | --- | --- | --- |
| Small HSB | Treatment 1 | Day 0-2 | 0.707 | 0.010 |
|  |  | Day 2-5 | 0.283 | 0.003 |
|  |  | Day 5-10 | 0.800 | <0.001 |
|  | Treatment 2 | Day 0-2 | 0.198 | 0.792 |
|  |  | Day 2-5 | 0.283 | 0.662 |
|  |  | Day 5-10 | 0.800 | 0.008 |

**C.**

| **Fish Size** | **Number of Injuries** | **Day Post Exposure** | **Diff of Means** | **P Values** |
| --- | --- | --- | --- | --- |
| Large HSB | Treatment 1 | Day 0 | 0.744 | <0.001 |
| vs. Small HSB |  | Day 2 | 0.594 | 0.013 |
|  |  | Day 5 | 1.233 | <0.001 |
|  |  | Day 10 | 0.455 | 0.073 |
|  | Treatment 2 | Day 0 | 1.048 | <0.001 |
|  |  | Day 2 | 0.393 | 0.088 |
|  |  | Day 5 | 0.017 | 0.944 |
|  |  | Day 10 | 0.333 | 0.155 |

**TABLE S4** Post-hoc Tukey test comparing individual treatments between the large hybrid striped bass and **A)** lake sturgeon, **B)** Chinook salmon, and **C)** Nile tilapia, from the 2 Way ANOVA analysis of RWI values for interaction of the different species and treatment levels.

**A.**

| **Large HSB vs. Lake Sturgeon** | **Diff of Means** | **P Values** |
| --- | --- | --- |
| 1 | 11.047 | <0.001 |
| 2 | 27.951 | <0.001 |
| 3 | 2.124 | 0.048 |
| 4 | 2.539 | 0.002 |

**B.**

| **Large HSB vs. Chinook Salmon** | **Diff of Means** | **P Values** |
| --- | --- | --- |
| 1 | 23.276 | <0.001 |
| 2 | 27.056 | <0.001 |
| 3 | 2.182 | 0.034 |
| 4 | 1.965 | 0.022 |

**C.**

| **Large HSB vs. Nile Tilapia** | **Diff of Means** | **P Values** |
| --- | --- | --- |
| 1 | 11.047 | <0.001 |
| 2 | 19.164 | <0.001 |
| 3 | 1.564 | 0.166 |
| 4 | 2.619 | 0.001 |

**TABLE S5** Post-hoc Tukey test comparing individual treatments between the large hybrid striped bass and **A)** lake sturgeon, **B)** Chinook salmon, and **C)** Nile tilapia, from the 2 Way ANOVA analysis of number of injuries observed for interaction of the different species and treatment levels.

**A.**

| **Large HSB vs. Lake Sturgeon** | **Diff of Means** | **P Values** |
| --- | --- | --- |
| 1 | 0.424 | 0.183 |
| 2 | 1.457 | <0.001 |
| 3 | 0.408 | 0.245 |
| 4 | 0.840 | <0.001 |

**B.**

| **Large HSB vs. Chinook Salmon** | **Diff of Means** | **P Values** |
| --- | --- | --- |
| 1 | 1.131 | <0.001 |
| 2 | 1.213 | <0.001 |
| 3 | 0.825 | <0.001 |
| 4 | 0.648 | 0.003 |

**C.**

| **Large HSB vs. Nile Tilapia** | **Diff of Means** | **P Values** |
| --- | --- | --- |
| 1 | 0.093 | 0.965 |
| 2 | 0.335 | 0.339 |
| 3 | 0.500 | 0.074 |
| 4 | 1.016 | <0.001 |
